# Thoracoscopic Transthoracic Hepatectomy for Hepatocellular Carcinoma in Budd‐Chiari Syndrome

**DOI:** 10.1111/ases.70101

**Published:** 2025-06-16

**Authors:** Atsushi Takebe, Masaya Nakano, Katsuya Ami, Chihoko Nobori, Yuta Yoshida, Takaaki Murase, Keiko Kamei, Ippei Matsumoto

**Affiliations:** ^1^ Department of Surgery Kindai University Hospital Osaka Japan

**Keywords:** Budd‐Chiari syndrome, hepatocellular carcinoma, transthoracic hepatectomy

## Abstract

Budd‐Chiari syndrome (BCS), caused by venous outflow obstruction, results in hepatic congestion and portal hypertension. BCS is also associated with a relatively high incidence of hepatocellular carcinoma (HCC). Selecting a minimally invasive approach based on hemodynamic assessment for the management of HCC arising from BCS is essential. A hepatic tumor located in liver segment 8 region was identified in an 88‐year‐old female patient with BCS. Following a detailed preoperative hemodynamic evaluation using angiography, a thoracoscopic transthoracic hepatectomy (TTH) was successfully performed. TTH may represent a feasible and effective surgical option for HCC in high‐risk patients, including those with BCS.

## 1 Introduction

Budd‐Chiari syndrome (BCS) is a rare condition characterized by hepatic congestion and portal hypertension caused by impaired hepatic venous outflow [1]. With progression, liver dysfunction and cirrhosis may develop [1]. Several types are based on the site and underlying cause of the venous obstruction [2]. Although a relatively high incidence of hepatocellular carcinoma (HCC) arising from chronic liver damage has been reported, the exact rate of carcinogenesis remains unclear [3]. Treating HCC associated with BCS requires an accurate evaluation of hepatic hemodynamics and residual liver function. Thoracoscopic transthoracic hepatectomy (TTH) for hepatic tumors is considered less invasive than transabdominal resection, particularly in selected cases, as it avoids liver mobilization. Although its indications are limited, this approach has been reported as a minimally invasive liver resection technique with favorable outcomes [4]. This report presents a patient with HCC that developed in the setting of cirrhosis secondary to BCS who underwent TTH.

## 2 Case Presentation

**FIGURE 1:**
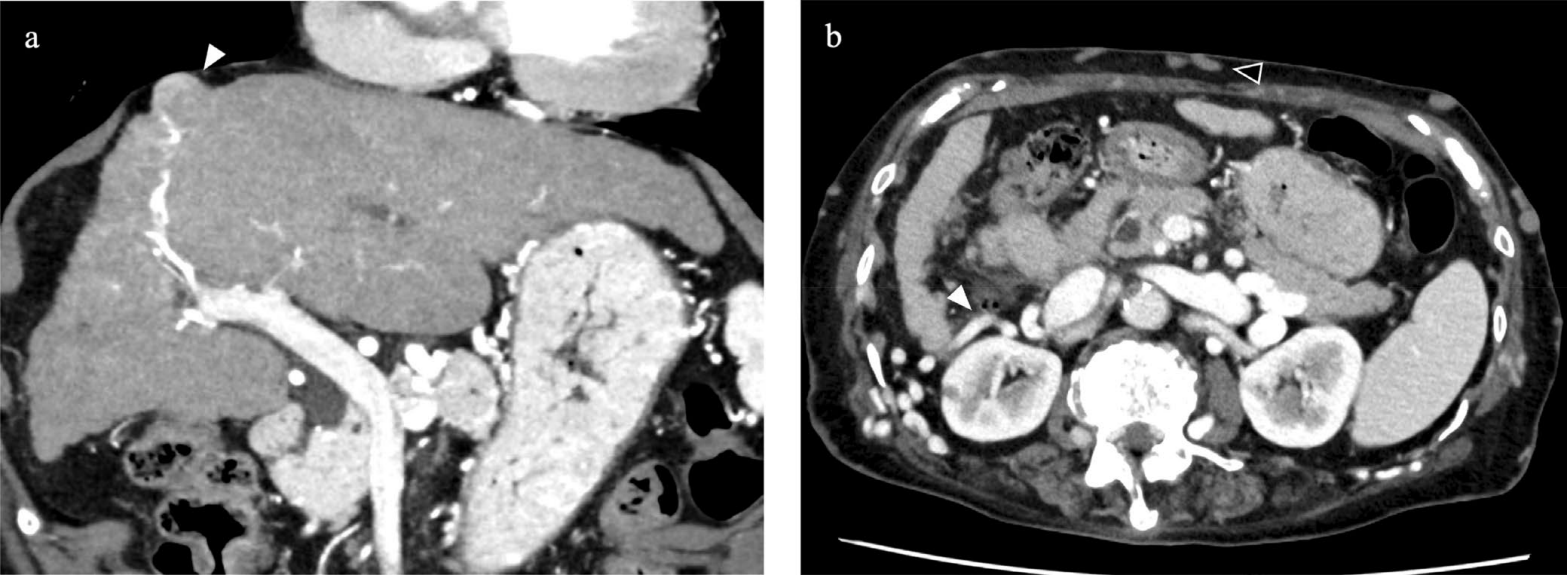
E‐CT examinations revealed a small tumor on the surface of liver segment 8 (white arrowhead). The liver morphology was suggestive of cirrhosis (a). Well‐developed collateral vessels were identified in the retroperitoneum (white arrowhead) and the abdominal wall (black arrowhead) (b).

**FIGURE 2:**
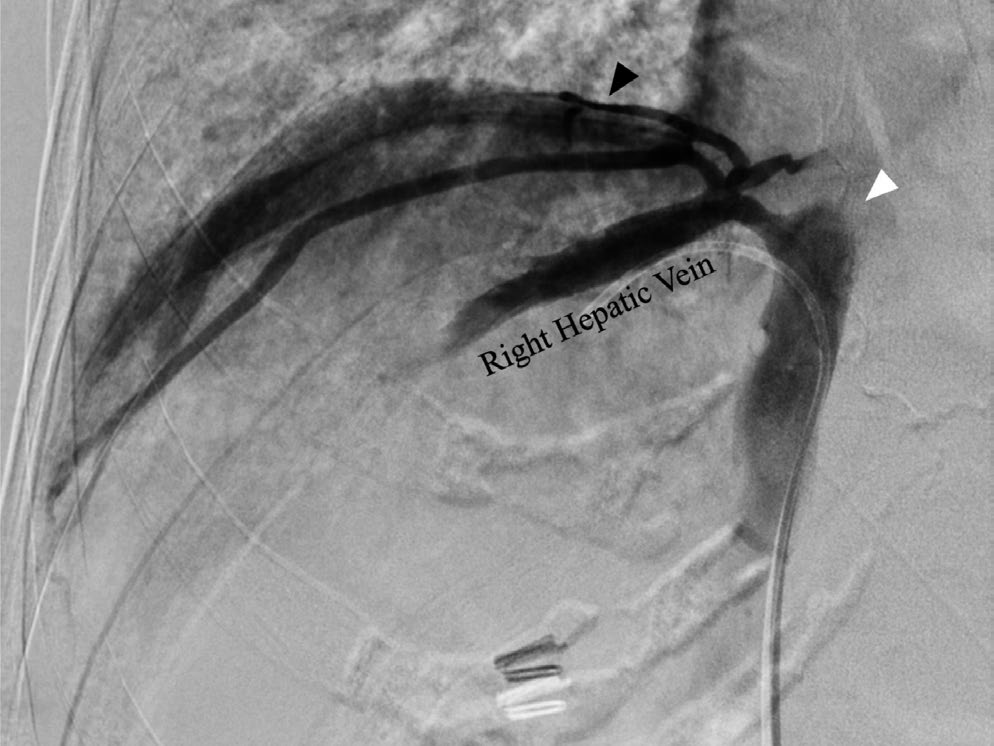
Cavography revealed severe stenosis of the suprahepatic IVC (white arrowhead) and developed collateral vessels (black arrowhead).

An 88‐year‐old woman was undergoing regular follow‐up for BCS suspected on preoperative examinations and intraoperative findings during laparoscopic cholecystectomy. Detailed examinations were performed due to elevated serum protein induced by vitamin K absence or antagonist‐II levels (109 mAU/mL), and a small nodule protruding from liver segment 8 was identified. The blood test results indicated liver dysfunction with a serum albumin level of 4.3 g/dL, a platelet count of 85 000/μL, and INR of 1.12. The 15‐min indocyanine green (ICG) clearance test value was 15%, suggesting that a minor liver resection would be feasible. Abdominal contrast‐enhanced computed tomography (CT) revealed a solitary tumor ~2 cm in diameter in liver segment 8 (Figure 1a). Morphologically, liver cirrhosis was evident, and collateral vessels were identified around the liver, retroperitoneum, abdominal wall, and chest wall (Figure 1b). TTH was planned as radiofrequency ablation (RFA) therapy was deemed unsuitable. Seven days preoperatively, cavography was performed to assess liver hemodynamics. Severe stenosis of the suprahepatic inferior vena cava and the presence of collateral vessels were observed (Figure 2). Although the inferior vena cava pressure was normal and no pressure gradient was observed in the right hepatic vein, balloon dilation of the stenotic area was additionally performed due to the potential destruction of collateral veins during surgery and the increased circulatory blood volume in the perioperative period (Figure S1).

**FIGURE 3:**
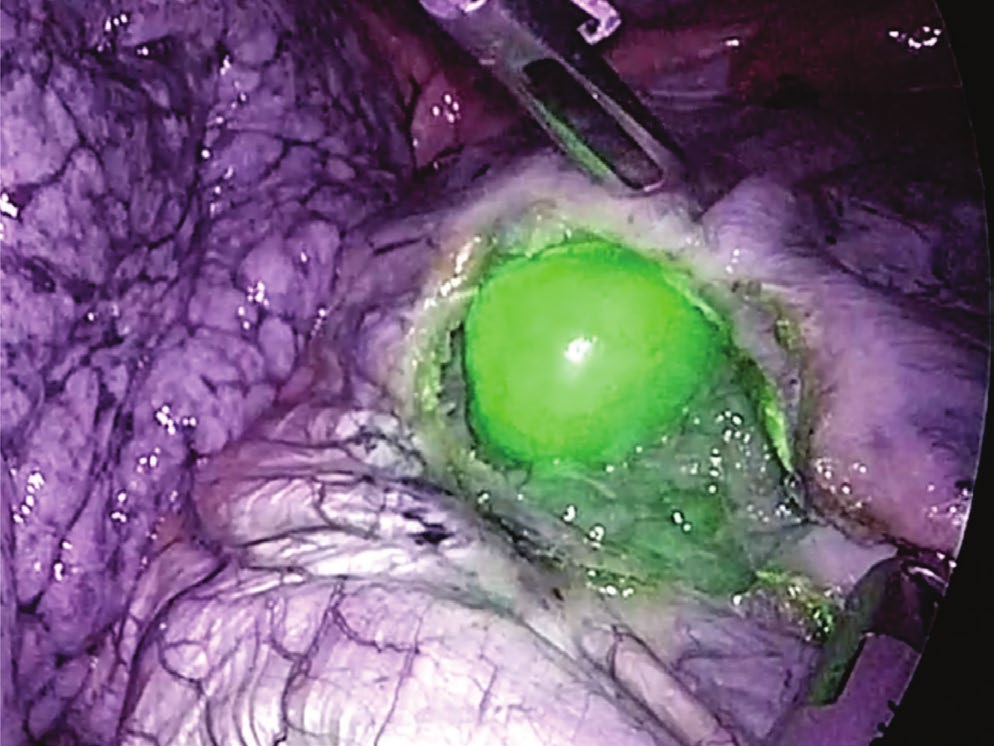
The tumor location was visualized through the diaphragm using ICG navigation. The diaphragm was opened in the minimal amount required.

On the day before surgery, 0.5 mg of ICG per kilogram of body weight was administered intravenously for ICG‐guided surgery. The patient was secured in the left lateral decubitus position, and the operation was started under general anesthesia with separate lung ventilation. A camera port was placed in the ninth intercostal space dorsally, and the thoracic cavity was inspected. Three 5 mm ports (2 for the surgeon and 1 for the assistant) were placed in the 7th and 10th intercostal spaces. Using the ICG observation mode, the tumor location was identified through the diaphragm. A minimal incision on the diaphragm was made after confirming there were no collateral vessels on the diaphragm directly above the tumor (Figure 3). Under an artificial pneumothorax (8 mmHg), partial hepatectomy was performed. The clamp‐crush method was used for parenchymal transection, with minimal bleeding from the transected surface. The diaphragm was simply repaired, and the surgery was completed (surgical procedure shown in Video S2). The operative time was 150 min, with minimal intraoperative bleeding. The patient was discharged to home on postoperative day 10 without complications. Pathological examination confirmed hepatocellular carcinoma with sufficient surgical margins. The background liver demonstrated F4‐grade fibrosis.

## 3 Discussion

In BCS, chronic liver damage occurs due to impaired hepatic blood flow caused by various factors. Treatment options include anticoagulant therapy, endovascular intervention, creation of a portosystemic shunt, surgical venoplasty, and liver transplantation [1]. Hepatic congestion is often relieved by shunting blood flow to the systemic circulation through collateral vessels. However, a detailed hemodynamic evaluation is essential for selecting the optimal treatment strategy. The patient had already developed liver cirrhosis and exhibited well‐developed collateral circulation; preoperative angiography and angioplasty were performed to address concerns about the risk of venous bleeding during hepatic dissection, which could result from abnormally high pressure in the hepatic veins.

In our case, we selected TTH, considering that liver mobilization would be highly invasive due to the collateral vessels from BCS and previous surgical history. TTH is mainly performed for small nodules in liver segment 8, particularly in patients with a history of upper abdominal surgery, and has demonstrated favorable outcomes in previous reports [4]. A major challenge of this surgical approach is the inability to control hepatic inflow using the Pringle maneuver. Therefore, there have been reports of utilizing pre‐coagulation techniques such as RFA [5]. Safe parenchymal transection was achieved in this case only using the conventional clamp‐crush method. One advantage of laparoscopic surgery is the reduction of venous bleeding due to the pressure from pneumoperitoneum [6]. In this case, liver parenchymal transection was performed under artificial pneumothorax with 8 mmHg pressure, which might contribute to bleeding control. Excessive intra‐abdominal pressure caused by the influx of CO_2_ gas into the abdominal cavity may rarely occur; careful monitoring of intraoperative condition is needed. Although indications for TTH are not yet established, we perform this procedure based on reported criteria [7]: tumors in the posterosuperior liver segments; multiple risks related to liver mobilization; and cases requiring combined liver and lung resection. We currently limit indications to small, superficial nodules that can be safely resected without the Pringle maneuver. To further improve surgical accuracy, we plan to perform robot‐assisted surgeries in the future.

The ICG‐guided liver surgery not only facilitates the identification of small nodules through preoperative administration but also enables the visualization of anatomical boundaries through intraoperative administration [8]. One of the challenges of transthoracic liver resection is identifying the tumor's location, and the usefulness of preoperative simulation using 3D CT imaging has also been reported [9]. In our case, the tumor location was easily identified even through the diaphragm, demonstrating the feasibility of ICG navigation in transthoracic liver resection.

## 4 Conclusion

Here we report successful minimally invasive surgery for HCC in an elderly cirrhotic patient with BCS, guided by detailed hemodynamic assessment. Although this condition is rare, we hope this report will contribute to the future management of liver diseases.

## Author Contributions

We confirm that the manuscript has been read and approved by all named authors.

## Ethics Statement

Informed consent was obtained from the patient for the use of clinical data.

## Conflicts of Interest

Dr. Ippei Matsumoto is an Editorial Board member of ASES Journal and a co‐author of this article. To minimize bias, they were excluded from all editorial decision‐making related to the acceptance of this article for publication. The other authors declare no conflicts of interest.

## Supporting information


**Figure S1:** Balloon angioplasty was performed on the stenotic area. Enhanced blood flow was observed following the intervention.

**Video S2:** Digest movie of surgical procedure.

## Data Availability

The data that supports the findings of this study are available in the supplementary material of this article.
