# Supplementary figures and images for: Thoracoscopic Transthoracic Hepatectomy for Hepatocellular Carcinoma in Budd‐Chiari Syndrome
Source: Asian J Endosc Surg. 2025 Jun 16;18(1):e70101. doi: 10.1111/ases.70101 (PMC12170063; doi:10.1111/ases.70101)

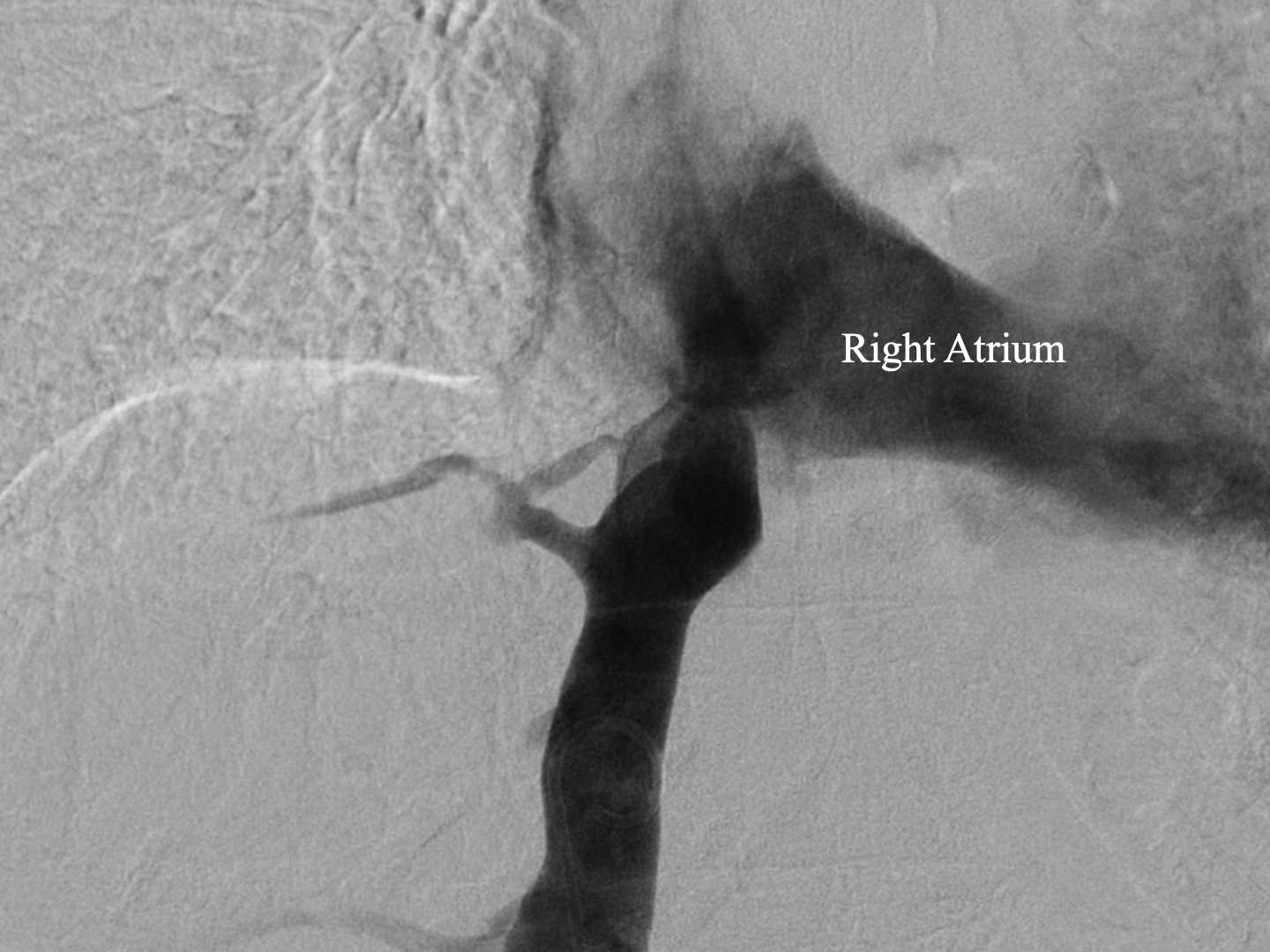

Supplement: Supplementary file 1 — Figure S1: Balloon angioplasty was performed on the stenotic area. Enhanced blood flow was observed following the intervention. [file ASES-18-e70101-s001.tiff]
